# Self-Reported Parosmia, Phantosmia, and Gustatory Dysfunction Among Adults with Mild-to-Moderate COVID-19: A Cross-Sectional Study in Saudi Arabia

**DOI:** 10.3390/clinpract15090167

**Published:** 2025-09-15

**Authors:** Reem A. Alsaqer, Ghazal Y. Dhaher, Rewa L. Alsharif, Razan Y. Almleaky, Khalid S. Menshawi, Turki M. Alqurashi, Abdullah Almaqhawi

**Affiliations:** 1 College of Medicine, King Faisal University, Al Hofuf 31982, Saudi Arabia; reemoo3111@gmail.com; 2 College of Medicine, King Abdulaziz University, Jeddah 80215, Saudi Arabia; ghazldh7@gmail.com (G.Y.D.); alqurashirazan@gmail.com (R.Y.A.); khalid_menshawi@hotmail.com (K.S.M.); 3 College of Medicine, King Saud Bin Abdulaziz University for Health Sciences, Jeddah 11481, Saudi Arabia; ralsharif313@gmail.com (R.L.A.); turkialqurashi778@gmail.com (T.M.A.); 4 Department of Family and Community Medicine, College of Medicine, King Faisal University, Al Hofuf 31982, Saudi Arabia

**Keywords:** COVID-19, SARS-CoV-2, parosmia, phantosmia, olfactory dysfunction

## Abstract

**Background/Objectives**: COVID-19 frequently causes olfactory and gustatory dysfunction, including qualitative disorders like parosmia and phantosmia. These distortions affect quality of life and may result from both peripheral and central neural damage. Despite increasing reports, their prevalence, mechanisms, and risk factors remain unclear. The purpose of this study is to evaluate the clinical characteristics and possible predictors of parosmia and phantosmia associated with COVID-19 in Saudi Arabia. **Methods**: This cross-sectional study utilized an online questionnaire targeting adults in Saudi Arabia with self-reported new-onset olfactory or gustatory dysfunction after COVID-19. **Results**: Out of 539 participants, 377 were included for analysis. Females slightly outnumbered males (195, 51.7% vs. 182, 48.3%) with a mean age of 34.5 years (SD = 12.7). Comorbidities were present in 86 (23.3%) participants, predominantly including hypertension (39.5%) and diabetes (30.2%). Sudden smell and taste loss were reported by 277 (73.5%) and 267 (70.8%) participants, respectively. Regional residence was significantly associated with both smell (*p* < 0.001) and taste loss (*p* < 0.001). Academic qualification exhibited borderline significance in relation to taste loss (*p* = 0.049). Logistic regression analysis indicated no significant predictors of dysfunction, with male gender exhibiting an odds ratio of 1.276 for smell (*p* = 0.301) and an odds ratio of 1.401 for taste (*p* = 0.144). Over 60% of participants experienced a negative impact on their quality of life. **Conclusions**: This study demonstrates the prevalence of parosmia and phantosmia in COVID-19 patients in Saudi Arabia, with a significant impact on quality of life. While regional differences and education level exhibited certain associations, no demographic or clinical factors independently predicted dysfunction, highlighting the necessity for additional research into underlying mechanisms and long-term effects.

## 1. Introduction

COVID-19-related olfactory dysfunction, which can occur alone or in combination with other respiratory symptoms, is becoming more well-known [1,2,3,4]. The presence of angiotensin converting enzyme-2 protein in sustentacular and olfactory stem cells makes the olfactory epithelium a target for the SARS-CoV-2 virus [5]. Olfactory disorders can be classified either as quantitative when odor sensitivity is affected (i.e., hyposmia/anosmia) or qualitative olfactory disorders (parosmia, phantosmia, or olfactory hallucinations) [6]. The most common variants of olfactory disorders in COVID-19 are hyposmia or sudden onset of anosmia [7,8]. However, there is an increasing number of reports in the literature of cases of parosmia and phantosmia associated with COVID-19 infection, implying that central olfactory pathways, in addition to the primary olfactory epithelium, are involved [9,10]. Parosmia is defined as a distorted perception of a true odor, whereas phantosmia is defined as an odor sensation without an odor source [6,11,12]. These olfactory disorders can have a negative impact on a person’s quality of life [13].

Furthermore, some studies have found that olfactory distortion causes more discomfort in patients than anosmia, or the isolated loss of the sense of smell [11,14]. However, parosmia is more commonly described in the literature as occurring during recovery in patients with postinfectious olfactory dysfunction [12,15,16]. Phantosmia is categorized into three types: cacosmic (associated with rotting, rotted, or fecal odors), torquosmic (linked to burnt, metallic, or chemical scents), or mixed (a combination of cacosmia and torquosmia) [17]. Phantosmia is classified into primary and secondary types. In this framework, primary phantosmia refers to an odor experienced without any discernible stimulus or underlying clinical condition, while secondary phantosmia denotes the perception of an odor without an evident stimulus that is linked to a recognized clinical disease process [17]. The pathogenesis of parosmia/phantosmia is currently unknown. According to one theory, the development of qualitative olfactory disorders is caused by a partial loss of olfactory receptors, resulting in insufficient formation of a typical odor-specific pattern in the olfactory bulb [11].

Another idea posits that parosmia results from atypical odor processing inside the central nervous system, specifically in the olfactory bulb [18,19], and/or in integrative brain areas associated with the olfactory system [20]. A diminished olfactory bulb volume in individuals with parosmia may signify a decrease in the quantity of bulb interneurons, leading to diminished lateral inhibition [21]. This causes an atypical pattern of olfactory activation, which modifies olfactory perception [22]. Prior studies have indicated that phantosmia originates in the peripheral olfactory neural system and/or the central nervous system [23].

Quantitative variants of olfactory disorder can be easily assessed using olfactory function tests, such as ‘Sniffin’ Sticks’ or the Smell Identification Test (UPSIT). While objective tools for qualitative olfactory dysfunction, such as the SSParoT (Sniffin’ Sticks Parosmia Test), do exist, they are less commonly applied in practice and research compared to quantitative assessments [13]. Despite numerous studies indicating consistent evidence of olfactory and gustatory dysfunction in COVID-19 patients, significant gaps persist concerning accurate prevalence estimates across various populations, symptom duration, recovery paths, and related risk factors. These uncertainties underscore the necessity for additional research, especially in under-represented areas like Saudi Arabia.

This study aimed to evaluate new-onset olfactory and gustatory dysfunction in adults with mild-to-moderate COVID-19 in Saudi Arabia, employing a specifically designed questionnaire. Its primary goal is to characterize the clinical aspects of parosmia and phantosmia in those who have COVID-19-related smell and taste problems. The study aims to assess the prevalence and examine patterns of olfactory and gustatory dysfunction in individuals infected with SARS-CoV-2.

## 2. Materials and Methods

### 2.1. Study Design and Setting

This is a descriptive cross-sectional study conducted across all five regions of Saudi Arabia (Eastern, Western, Central, Northern, and Southern). A bespoke, validated online questionnaire was disseminated via social media and healthcare networks to reach adults who experienced laboratory-confirmed mild-to-moderate COVID-19 and self-reported new-onset olfactory or gustatory dysfunction.

### 2.2. Participants

Eligible participants were Saudi residents aged ≥ 18 years (men and non-pregnant women) who had confirmed COVID-19 and newly developed parosmia or phantosmia. Exclusion criteria included individuals who were not residents in Saudi Arabia, aged < 18, pregnant, did not contract COVID-19, and had no pre-existing chemosensory disorders.

### 2.3. Sampling and Sample Size

A convenience sampling method was employed, leveraging online platforms to recruit approximately 400–500 participants to ensure sufficient power for regional subgroup analysis. Sample size calculations were based on expected prevalence rates and logistic regression power requirements [24,25]. A total of 377 eligible participants were included in the analysis due to constraints in resources and recruitment. A pilot study involving 10 participants from the target population was conducted to evaluate face validity. Before the primary survey, the questionnaire was improved based on input on item relevance, comprehensibility, and clarity. Olfactory and gustatory dysfunction following COVID-19 is characterized by symptoms that emerged during the acute infection and continued beyond this phase. Participants who experienced new dysfunction solely after achieving clinical recovery from COVID-19 were excluded from this category.

### 2.4. Data Collection Instrument

The questionnaire was originally developed and validated in English [26]. For the purposes of this study, we performed a forward–backward translation process to Arabic. The forward translation was performed by two bilingual experts, and the accuracy of the Arabic version was validated through back-translation into English. The prefinal version underwent review by three senior consultants to evaluate content clarity and cultural appropriateness. A pilot test was conducted involving 10 participants to assess comprehensibility and ease of completion. No substantial modifications were applied to the original items aside from linguistic adjustments. The following variables were examined:

- Demographics and clinical history: age, sex, marital status, education, occupation, comorbidities, and COVID-19 confirmation.
- Smell and taste dysfunction: onset, severity, and persistence of parosmia/phantosmia versus anosmia/hyposmia.
- Symptom categorization: whether dysfunction occurred during acute illness and if it was still active at survey completion.
- Quality-of-life impact: influence on daily functioning and well-being.
- Regional and educational effects: to explore potential predictors.

The questionnaire underwent pilot testing for clarity and face validity prior to full deployment. Participants were allowed to report more than one comorbidity; however, for the purpose of analysis, we categorized them based on the most significant condition reported. Definitions used were as follows: “Alcohol use” refers to self-reported consumption of alcohol, not a clinical diagnosis of alcohol use disorder; “Heart disease” includes ischemic heart disease as well as other cardiovascular conditions; and “SCD” refers specifically to sickle cell disease. The category “Other” includes less common comorbidities (e.g., hypothyroidism, autoimmune conditions such as rheumatoid arthritis, and other isolated disorders), which were grouped together due to their low frequency.

### 2.5. Study Timeline

Data collection occurred over a four-month duration, from January 2025 to April 2025. Participants provided informed consent electronically before completing the questionnaire.

### 2.6. Data Management

Responses were extracted into SPSS v29.0.0 and stored securely without any personal identifiers (e.g., name or ID), ensuring participants’ anonymity.

### 2.7. Statistical Analysis

Descriptive statistics summarized demographic and clinical variables. Associations between categorical variables (e.g., region, education, and persistent dysfunction) were tested using chi-square or Fisher’s exact tests. To identify adjusted predictors of smell and taste dysfunction, binary logistic regression models were constructed. Binary logistic regression models were developed to determine adjusted predictors of ongoing smell and taste dysfunction. The models were adjusted for pre-selected demographic and clinical variables identified as potential confounders in the literature: gender, age, social status, nationality, and the presence of comorbidities. All analyses were conducted in SPSS v29.0.0, with significance set at *p* < 0.05.

## 3. Results

We received 539 participant responses for the assessment of clinical features of parosmia/phantosmia associated with COVID-19 infection in Saudi Arabia, but only 377 were analyzed based on inclusion criteria, while the remaining were excluded based on exclusion criteria (Table 1). The gender distribution was nearly balanced, with females comprising slightly more than half (*n* = 195, 51.7%) and males comprising slightly less than half (*n* = 182, 48.3%). The mean age was 34.5 years (SD = 12.7), ranging from 18 to 67 years. Most participants were married (*n* = 200, 53.1%), followed by single participants (*n* = 156, 41.4%) and widowed/divorced individuals (*n* = 21, 5.6%). The majority were Saudi nationals (*n* = 340, 90.2%), with the Eastern region being the most represented area (*n* = 162, 43.0%). Educational levels were high, with most holding a bachelor’s degree (*n* = 226, 59.9%). Employment was reported by 45.1% (*n* = 170), while 25.5% (*n* = 96) were students. A minority reported comorbidities (*n* = 86, 23.3%), while the remaining participants had no known conditions (*n* = 283, 76.7%).

**Figure 1:**
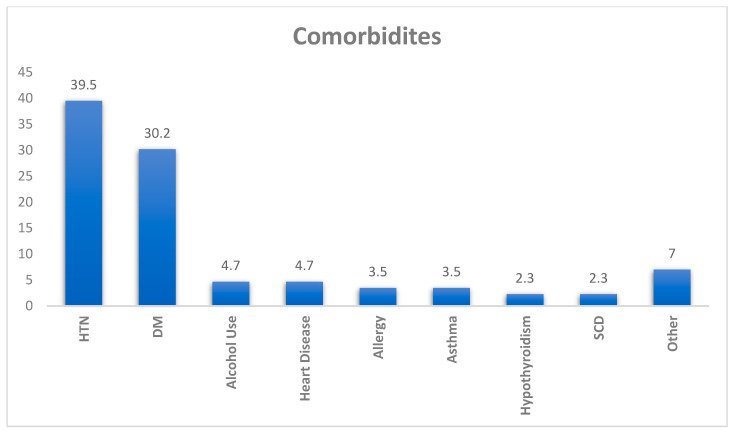
Different comorbidities among participants (*n* = 86).

Figure 1 shows the distribution of comorbidities among participants with existing health conditions (*n* = 86). The most prevalent comorbidity was hypertension (HTN), reported by 39.5% of participants, followed by diabetes mellitus (DM) at 30.2%. Less commonly reported conditions included alcohol use and heart disease (each 4.7%), allergy, and asthma. Other comorbidities like hypothyroidism, rheumatoid arthritis (RA), and sickle cell disease (SCD) were reported by 2.3% of the participants each. The category labeled “Other” accounted for 7.0%, suggesting additional unspecified conditions.

Table 2 shows the clinical features associated with parosmia and phantosmia among patients experiencing COVID-19-related olfactory and gustatory dysfunction (*n* = 377). A vast majority experienced a sudden onset of smell (*n* = 277, 73.5%) and taste loss (*n* = 267, 70.8%) during their infection, indicating acute sensory disruption. Beyond mere loss, altered perception was highly prevalent, with nearly 7 in 10 participants (68.4%) reporting distorted smells and 65.5% reporting taste changes, suggesting that sensory quality, not just intensity, was affected. Psychosocial and functional impacts were pronounced. About 45% of respondents reported feeling isolated due to smell loss, and 40.8% due to taste loss, pointing to the strong social withdrawal that these dysfunctions can provoke. Importantly, around 4 in 10 participants (41.1% for smell and 39.3% for taste) stated that their daily functioning was disrupted, underlining the real-world limitations imposed by these changes. Emotional responses were widespread: nearly half of the cohort (48.0% and 50.7% for smell and taste, respectively) felt anger as a result of sensory alterations. Dietary behaviors were also significantly altered, with over half changing their food intake due to smell (56.8%) and taste (61.3%) changes. Perhaps most telling, around two-thirds reported a diminished enjoyment of food and drink (64.2% for smell and 65.0% for taste), reflecting a tangible decline in quality of life. Finally, nearly half of the participants (49.9% for smell and 47.5% for taste) stated they had to exert more effort to relax, indicating a broader psychological toll beyond the sensory symptoms.

Table 3 shows the association between post-COVID-19 olfactory dysfunction (loss of smell) and various sociodemographic and clinical characteristics among 377 participants. Loss of smell after COVID-19 was more frequently reported by females (*n* = 139, 71.3%) than males (*n* = 138, 75.8%), but the association was not statistically significant (*p* = 0.318). The mean age was similar between those with and without post-COVID-19 smell loss (34.6 vs. 34.4 years, *p* = 0.912), indicating no age-related difference. Marital status, nationality, education level, occupation, and comorbidity status were also not significantly associated with post-COVID-19 olfactory dysfunction (*p* > 0.05 in all cases). However, the place of residence showed a statistically significant association (*p* < 0.001). The Central (92.9%) and Southern (94.4%) regions had the highest proportions of reported olfactory loss compared to the Eastern (71.0%) and Western (66.4%) regions.

Table 4 shows the association between gustatory dysfunction (loss of taste) following COVID-19 and various demographic and clinical features in a sample of 377 participants. Although a slightly higher proportion of males (*n* = 135, 74.2%) than females (*n* = 132, 67.7%) reported taste loss, this difference was not statistically significant (*p* = 0.166). The mean age was comparable between those with and without taste loss (34.0 vs. 35.5 years; *p* = 0.322). Marital status, nationality, occupation, and comorbidity status did not show significant associations with taste dysfunction (all *p* > 0.05). However, place of residence was significantly associated with taste loss (*p* < 0.001), with 100% of participants from the Northern and Southern regions reporting gustatory dysfunction, compared to 61.4% in the Western and 68.5% in the Eastern region. Additionally, academic qualification showed a borderline significant association (*p* = 0.049). All participants with education up to middle school (*n* = 12, 100%) reported taste loss, while rates were slightly lower among higher education levels. These findings suggest that regional and educational factors may influence the persistence or perception of taste dysfunction after COVID-19.

Table 5 shows the adjusted predictors of olfactory and gustatory dysfunction after COVID-19, using logistic regression analysis. None of the variables showed statistically significant associations (all *p* > 0.05), suggesting that these demographic and clinical factors were not strong independent predictors of persistent smell or taste loss after COVID-19. For smell loss, males had an adjusted odds ratio (OR) of 1.276 (95% CI: 0.804–2.024, *p* = 0.301), indicating a nonsignificant trend toward higher odds. Age, marital status, Saudi nationality, and comorbidities also did not significantly predict olfactory dysfunction. Similarly, for taste loss, male participants had 1.4 times the odds (OR = 1.401, 95% CI: 0.891–2.202, *p* = 0.144), but again, this was not significant. Other predictors, including age, marital status, nationality, and comorbidities, showed no meaningful associations (Table 6).

## 4. Discussion

This study revealed that a significant majority of participants experienced a sudden loss of smell (73.5%) and taste (70.8%) during COVID-19 infection. Additionally, notable proportions reported qualitative changes, including altered smell (68.4%) and taste (65.5%). These findings align with the global literature recognizing olfactory and gustatory dysfunction as key characteristics of COVID-19. Saniasiaya et al. (2021), in a meta-analysis of 27,492 patients, reported a 47.85% prevalence of olfactory dysfunction, supported by high-quality evidence [27]. Similarly, Gupta et al. (2021) found that 71% of patients experienced taste disorders [28]. Furthermore, Goyal et al. (2021) observed that 34.84% of patients reported alterations in smell, while 46.86% reported changes in taste following COVID-19 infection [29]. In addition to prevalence, our study highlights the psychosocial and functional impact of these symptoms, with nearly half of the participants reporting social isolation or challenges in daily activities due to sensory changes. The high prevalence of olfactory and gustatory dysfunction in our cohort reinforces the critical role that these sensory disruptions play in the clinical spectrum of COVID-19 and underscores the necessity of early identification and management in post-COVID-19 care.

Moreover, this study shows that the psychosocial and functional impacts were also significant, with approximately half of the participants feeling isolated or experiencing difficulties in daily activities due to altered smell or taste. Similarly, Menchero et al. (2024) show that there are cases of social isolation and a lack of interest in the activities of daily living, as a reaction of the patients to the persistence of problems with taste and smell after COVID-19 [30]. There are emotional disturbances such as anger and frustration, along with changes in dietary habits and the diminished enjoyment of food in patients. Similarly, a study by Watson et al. (2021) shows that there is a profound impact on the dietary behaviors, social interactions, and emotional well-being of participants after gustatory and olfactory dysfunction after COVID-19 [31]. Additionally, Stankevice et al. (2023) show that persistent taste and smell dysfunction significantly reduces quality of life, underscoring the importance of recognizing and addressing these symptoms clinically [32].

Interestingly, despite the substantial prevalence and impact observed, these analyses did not reveal significant demographic predictors for either the olfactory or the gustatory dysfunction. Specifically, the gender differences did not show a statistically significant association, but males showed a slightly higher proportion of taste dysfunction. This contrasts with some studies reporting female predominance in post-COVID-19 symptoms like sensory dysfunction, which suggests that there is a potential gender-specific vulnerability [33]. The study by Turk et al. (2022) indicates that the prevalence of ongoing smell and/or taste loss did not differ statistically between male and female patients [34]. The nonsignificant findings related to gender may be due to the balanced representation of male and female participants, which reduces the likelihood of detecting differences typically observed in studies with skewed gender ratios.

Notably, this study identified place of residence as a significant predictor for both olfactory and gustatory dysfunction. Participants from the Central and Southern regions reported higher rates of olfactory dysfunction, while all participants from the Northern and Southern regions reported gustatory dysfunction. These regional differences likely reflect variations in awareness, reporting tendencies, and perception of symptoms rather than true biological differences. Similar geographical disparities have been previously observed in the Saudi context, highlighting differences in health literacy and symptom recognition across regions [35].

Regarding education, our findings showed a borderline significant association between lower educational attainment and reported gustatory dysfunction. This may reflect differences in symptom awareness and reporting, as individuals with lower education levels may be more likely to notice or report noticeable sensory changes, rather than indicating delayed healthcare-seeking behavior [36]. These considerations suggest that self-reported data may be influenced by participants’ perception and awareness of symptoms and should be interpreted in this context.

Hypertension and diabetes were the most prevalent comorbidities, representing nearly two-thirds of all cases. Our regression analyses did not reveal significant associations between comorbidities and persistent sensory dysfunction. The previous literature indicates that chronic diseases may affect COVID-19 severity and sensory recovery [37]. However, the lack of significant findings in our study may result from the limited sample size of participants with comorbid conditions, highlighting the necessity for further research with larger cohorts. The logistic regression analysis did not identify any demographic or clinical variables that significantly predicted persistent olfactory or gustatory dysfunction in the sample studied.

There are several limitations of this study. The cross-sectional design of this study restricts the ability to establish the causal relationships between COVID-19 and the persistent olfactory or gustatory dysfunction. This study’s reliance on self-reported data may introduce recall bias or subjective misinterpretation of symptoms. The absence of objective olfactory assessment and taste testing (e.g., UPSIT) may diminish diagnostic accuracy. Finally, the uneven regional representation may influence the generalisability of the findings to the wider Saudi population.

Participants indicated a significant occurrence of parosmia and phantosmia post-COVID-19, underscoring possible effects on psychosocial well-being and daily activities. The findings indicate that clinicians should evaluate sensory disturbances within a comprehensive supportive care framework, which encompasses psychological support and dietary counseling, without suggesting population-level prevalence or necessitating routine screening.

Future research should adopt longitudinal designs to explore the duration and the recovery trajectory of the olfactory and gustatory dysfunction. The incorporation of objective diagnostic tools and neuroimaging may help to elucidate the underlying mechanisms. There is a need for regionally diverse samples, which is also essential to enhance generalizability and guide targeted public health interventions. Furthermore, recruitment through social media may result in selection bias, and self-reported COVID-19 diagnoses and sensory complaints lacking clinical verification diminish the accuracy and generalizability of the results. The study used a simple present/absent approach for olfactory and gustatory dysfunction without severity scales, limiting the assessment of symptom intensity. Also, our sample size calculation relied on prevalence rates reported in initial studies conducted at the start of the COVID-19 pandemic. These early estimates may differ from more recent data due to changes in viral variants, diagnostic practices, and population characteristics. This should be considered when interpreting the representativeness of our findings.

In addition, the impact of circulating SARS-CoV-2 variants during the study period should be taken into account when interpreting our results. Recent evidence indicates that various variants correlate with differing prevalence and severity of olfactory and gustatory dysfunctions. Due to insufficient regional sequencing data, we could not identify the dominant variant in Saudi Arabia during the recruitment period. The immunity profile of the population in 2025, influenced by extensive vaccination and previous infections, is likely distinct from that observed in earlier phases of the pandemic. These factors may partially account for the discrepancies between our findings and those reported in studies from 2020 to 2021 and should be taken into account when making temporal comparisons. The comorbidity data in this study were self-reported by participants and were not validated through clinical records. Furthermore, data regarding disease stage, severity, duration, and treatment were not obtained. These factors may affect olfactory and gustatory results and should be taken into account when analyzing the findings.

## 5. Conclusions

This study demonstrates that olfactory and gustatory dysfunction associated with COVID-19 has a significant effect on patients’ daily activities and emotional health. The analysis revealed no demographic or clinical variables that significantly predicted persistent dysfunction, underscoring the complex and multifactorial nature of post-COVID-19 sensory disturbances. Future research should prioritize longitudinal designs and incorporate biological markers alongside psychosocial measures to enhance our understanding and management of these outcomes.

## Figures and Tables

**Table 1:** Sociodemographic characteristics and other parameters of participants (*n* = 377).

		Frequency N (%)
Gender	Female	195 (51.7)
	Male	182 (48.3)
Age (Years)	Mean (Sd)	34.5 (12.7)
	Range	18–67
Marital Status	Widow/Divorced	21 (5.6)
	Single	156 (41.4)
	Married	200 (53.1)
Nationality	Non-Saudi	37 (9.8)
	Saudi	340 (90.2)
Residence	Eastern Region	162 (43.0)
	Western Region	140 (37.1)
	Northern Region	29 (7.7)
	Central Region	28 (7.4)
	Southern Region	18 (4.8)
Academic Qualification	Up to Middle School	12 (3.2)
	Secondary	85 (22.5)
	Bachelor’s	226 (59.9)
	Post-graduate	54 (14.3)
Occupation	Unemployed	60 (15.9)
	Student	96 (25.5)
	Employed	170 (45.1)
	Freelance Work	23 (6.1)
	Retired	28 (7.4)
Comorbidities	No	283 (76.7)
	Yes	86 (23.3)

**Table 2:** Assessment of clinical features of parosmia and phantosmia among coronavirus disease (COVID-19)-related olfactory and gustatory dysfunction patients. (*n* = 377).

	Frequency N (%)
Sudden loss of smell with COVID-19	277 (73.5)
Sudden loss of taste with COVID-19	267 (70.8)
Change in smell perception (beyond intensity)	258 (68.4)
Change in taste perception (beyond intensity)	247 (65.5)
Smell change makes you feel isolated	168 (44.6)
Taste change makes you feel isolated	154 (40.8)
Smell change causes difficulty in daily activities	155 (41.1)
Taste change causes difficulty in daily activities	148 (39.3)
Smell change makes you feel angry	181 (48.0)
Taste change makes you feel angry	191 (50.7)
Smell change alters eating amount	214 (56.8)
Taste change alters eating amount	231 (61.3)
Smell change reduces enjoyment of food/drink	242 (64.2)
Taste change reduces enjoyment of food/drink	245 (65.0)
Smell change prompts more effort to relax	188 (49.9)
Taste change prompts more effort to relax	179 (47.5)

**Table 3:** Association between olfactory dysfunctions after COVID-19 and different features (*n* = 377).

		Loss of Smell After COVID-19 Infection		Sig. Value
		No N (%)	Yes N (%)	
Gender	Female	56 (28.7)	139 (71.3)	0.318 a
	Male	44 (24.2)	138 (75.8)	
Age (Years)	Mean (Sd)	34.6 (13.1)	34.4 (12.5)	0.912 c
Marital Status	Widow/Divorced	4 (19.0)	17 (81.0)	0.462 a
	Single	46 (29.5)	110 (70.5)	
	Married	50 (25.0)	150 (75.0)	
Nationality	Non-Saudi	9 (24.3)	28 (75.7)	0.749 a
	Saudi	91 (26.8)	249 (73.2)	
Place of Residence	Eastern Region	47 (29.0)	115 (71.0)	<0.001 b
	Western Region	47 (33.6)	93 (66.4)	
	Northern Region	3 (10.3)	26 (89.7)	
	Central Region	2 (7.1)	26 (92.9)	
	Southern Region	1 (5.6)	17 (94.4)	
Academic Qualification	Up to Middle School	1 (8.3)	11 (91.7)	0.494 b
	Secondary	25 (29.4)	60 (70.6)	
	Bachelor’s	61 (27.0)	165 (73.0)	
	Post-graduate	13 (24.1)	41 (75.9)	
Occupation	Unemployed	18 (30.0)	42 (70.0)	0.387 a
	Student	27 (28.1)	69 (71.9)	
	Employed	42 (24.7)	128 (75.3)	
	Freelance	3 (13.0)	20 (87.0)	
	Retired	10 (35.7)	18 (64.3)	
Comorbidities	No	76 (26.1)	215 (73.9)	0.741 a
	Yes	24 (27.9)	62 (72.1)	

(a) Chi-Square Test, (b) Fisher’s Exact Test, and (c) Independent *T* Test.

**Table 4:** Association between gustatory dysfunctions after COVID-19 and different features (*n* = 377).

		Loss of Taste After COVID-19 Infection		Sig. Value
		No N (%)	Yes N (%)	
Gender	Female	63 (32.3)	132 (67.7)	0.166 a
	Male	47 (25.8)	135 (74.2)	
Age (Years)	Mean (Sd)	35.5 (12.9)	34.0 (12.6)	0.322 c
Marital Status	Widow/Divorced	4 (19.0)	17 (81.0)	0.488 a
	Single	44 (28.2)	112 (71.8)	
	Married	62 (31.0)	138 (69.0)	
Nationality	Non-Saudi	7 (18.9)	30 (81.1)	0.148 a
	Saudi	103 (30.3)	237 (69.7)	
Place of Residence	Eastern Region	51 (31.5)	111 (68.5)	<0.001 a
	Western Region	54 (38.6)	86 (61.4)	
	Northern Region	0 (0.0)	29 (100.0)	
	Central Region	5 (17.9)	23 (82.1)	
	Southern Region	0 (0.0)	18 (100.0)	
Academic Qualification	Up to Middle School	0 (0.0)	12 (100.0)	0.049 b
	Secondary	30 (35.3)	55 (64.7)	
	Bachelor’s	66 (29.2)	160 (70.8)	
	Post-graduate	14 (25.9)	40 (74.1)	
Occupation	Unemployed	20 (33.3)	40 (66.7)	0.421 a
	Student	25 (26.0)	71 (74.0)	
	Employed	50 (29.4)	120 (70.6)	
	Freelance	4 (17.4)	19 (82.6)	
	Retired	11 (39.3)	17 (60.7)	
Comorbidities	No	80 (27.5)	211 (72.5)	0.185 a
	Yes	30 (34.9)	56 (65.1)	

(a) Chi-Square Test, (b) Fisher’s Exact Test, and (c) Independent *T* Test.

**Table 5:** Adjusted predictors of olfactory dysfunctions after COVID-19 (*n* = 377).

	Sig.	*p*-Value	Adjusted Odds Ratio (aOR)	95% CI	
				Lower	Upper
Gender (Male)	0.244	0.301	1.276	0.804	2.024
Age	−0.001	0.943	0.999	0.979	1.020
Social Status (Married vs. Single)	0.083	0.686	1.086	0.727	1.622
Nationality (Saudi)	−0.154	0.704	0.857	0.387	1.898
Comorbidities (Present)	−0.114	0.702	0.892	0.497	1.600
Constant	0.974	0.070	2.649		

Multivariable Logistic Regression Analysis of Predictors for Persistent Olfactory Dysfunction.

**Table 6:** Adjusted predictors of gustatory dysfunctions after COVID-19 (*n* = 377).

	Sig.	*p*-Value	Adjusted Odds Ratio (aOR)	95% CI	
				Lower	Upper
Gender (Male)	0.337	0.144	1.401	0.891	2.202
Age	−0.003	0.798	0.997	0.978	1.017
Social Status (Married vs. Single)	−0.130	0.529	0.878	0.587	1.315
Nationality (Saudi)	−0.620	0.159	0.538	0.227	1.275
Comorbidities (Present)	−0.327	0.251	0.721	0.412	1.261
Constant	1.658	0.003	5.248		

Multivariable Logistic Regression Analysis of Predictors for Persistent Gustatory Dysfunction.

## Data Availability

The data presented in this study are available upon reasonable request from the corresponding author. The data are not publicly available due to privacy or ethical restrictions.
